# Identification of genes and key pathways underlying the pathophysiological association between nonalcoholic fatty liver disease and atrial fibrillation

**DOI:** 10.1186/s12920-022-01300-1

**Published:** 2022-07-05

**Authors:** Yanan Chu, Fangcong Yu, Yakui Wu, Jinxiu Yang, Jiaran Shi, Tianxin Ye, Deheng Han, Xingxiang Wang

**Affiliations:** Department of Cardiology, The First Affiliated Hospital, School of Medicine, Zhejiang University, No. 79 Qingchun Road, Hangzhou, 310006 Zhejiang China

**Keywords:** Nonalcoholic fatty liver disease, Atrial fibrillation, Bioinformatic technology, Differentially expressed genes, Hub genes

## Abstract

**Background:**

Atrial fibrillation (AF) is one of the most prevalent sustained cardiac arrhythmias. The latest studies have revealed a tight correlation between nonalcoholic fatty liver disease (NAFLD) and AF. However, the exact molecular mechanisms underlying the association between NAFLD and AF remain unclear. The current research aimed to expound the genes and signaling pathways that are related to the mechanisms underlying the association between these two diseases.

**Materials and methods:**

NAFLD- and AF- related differentially expressed genes (DEGs) were identified via bioinformatic analysis of the Gene Expression Omnibus (GEO) datasets GSE63067 and GSE79768, respectively. Further enrichment analysis of Gene Ontology (GO) and Kyoto Encyclopedia of Genes and Genomes (KEGG), the construction of a protein–protein interaction (PPI) network, the identification of significant hub genes, and receiver operator characteristic curve analysis were conducted. The gene-disease interactions were analyzed using the Comparative Toxicogenomics Database. In addition, the hub genes were validated by quantitative Real-Time PCR (qRT-PCR) in NAFLD cell model.

**Results:**

A total of 45 co-expressed differentially expressed genes (co-DEGs) were identified between the NAFLD/AF and healthy control individuals. GO and KEGG pathway analyses revealed that the co-DEGs were mostly enriched in neutrophil activation involved in the immune response and cytokine-cytokine receptor interactions. Moreover, eight hub genes were selected owing to their high degree of connectivity and upregulation in both the NAFLD and AF datasets. These genes included *CCR2*, *PTPRC*, *CXCR2*, *MNDA*, *S100A9*, *NCF2*, *S100A12*, and *S100A8*.

**Conclusions:**

In summary, we conducted the gene differential expression analysis, functional enrichment analysis, and PPI analysis of DEGs in AF and NAFLD, which provides novel insights into the identification of potential biomarkers and valuable therapeutic leads for AF and NAFLD.

**Supplementary Information:**

The online version contains supplementary material available at 10.1186/s12920-022-01300-1.

## Introduction

Atrial fibrillation (AF) is characterized by left atrial electrical and structural reconstruction, and is the most frequent type of perpetual arrhythmia among the general population [1]. Due to irregular electronic activity and abnormal blood flow, ischemic stroke thromboembolism originating from the left atrium is the most common cardiovascular system disease. AF affects over 33 million people worldwide, and the incidence of AF continues to increase [2, 3]. The growing incidence of AF is associated with several risk factors including obesity, smoking, hypertension, diabetes, coronary artery disease and heart failure [4]. Moreover, increasing evidence indicates the role of inflammation in the pathophysiology of AF [5]. However, current treatments for AF are limited in their effects. Thus, this disorder is a major health problem worldwide and is associated with substantially increased morbidity, mortality, and health care expenditures [6].

Nonalcoholic fatty liver disease (NAFLD) is defined as the excessive accumulation of fat in liver cells, accounting for at least 5% of liver weight, without excessive alcohol intake [7]. Approximately 15%-30% of adults are affected with NAFLD, and the prevalence is increased in obese or diabetic populations [8]. NAFLD has become the predominant cause of liver failure and hepatocellular carcinoma in many countries worldwide [9]. In addition to liver disease, accumulating evidence indicates that NAFLD is also strongly associated with cardiovascular diseases; it adversely affects not only coronary artery disease but also cardiac arrhythmias [10]. The existence of a possible association between NAFLD and the risk of progression of AF has been a focus of scientific investigations, and a number of clinical studies have shown that NAFLD is very common in patients with AF. The Framingham Heart Study research fellows have reported a close link between NAFLD and an increased risk for the onset of AF in the community [11]. Targher et al. reported that patients with NAFLD had a higher prevalence of AF than subjects without NAFLD [12]. NAFLD and AF share some similar risk factors and morbidities, including obesity, type 2 diabetes, and metabolic syndrome [13]. NAFLD is associated with obesity, autonomic dysfunction, and systemic inflammation. All these conditions are also risk factors for AF [14]. The possible effect of NAFLD on the onset and progression of AF is worth special concern, and the potential mechanistic links between NAFLD and AF are still unclear [15].

Due to the development of the second-generation sequencing technology, advances in molecular biology, and the establishment of a number of algorithms, bioinformatic analysis has been comprehensively applied to explore the underlying mechanisms of gene networks. The networks, provide comprehensive insights into the pathogenic mechanisms or therapeutic assessment of multiple diseases, including NAFLD and AF [16–18]. Bioinformatics analysis can obtain a large number of gene expression patterns and explore differentially expressed genes (DEGs) related to disease initiation and progression [19]. Hence, Zhang et al. provided new evidence for the underlying mechanism of AF complicated with stroke by using bioinformatics analyses [20]. Recently, a few diagnostic biomarkers for NAFLD and AF have been found by bioinformatic analysis [18, 21], but the genetic relationships between NAFLD and AF are poorly studied.

In the current study, the gene expression datasets for NAFLD (GSE63067) and AF (GSE79768) were downloaded from the Gene Expression Omnibus (GEO, http://www.ncbi.nlm.nih.gov/geo/) database, which is a public genomics data repository that provides genetic information on diseases [22]. Co-expressed differentially expressed genes (co-DEGs) of NAFLD and persistent AF were found, and the molecular mechanisms of the co-DEGs were examined via Gene Ontology (GO) term enrichment, Kyoto Encyclopedia of Genes and Genomes (KEGG), and protein–protein interaction (PPI) network analyses. Finally, the diagnostic information of the hub genes was investigated, and the possible value of these hub genes as a new therapeutic target for patients with NAFLD and AF is discussed.

## Materials and Methods

### NAFLD and AF microarray dataset collection

**Fig. 1:**
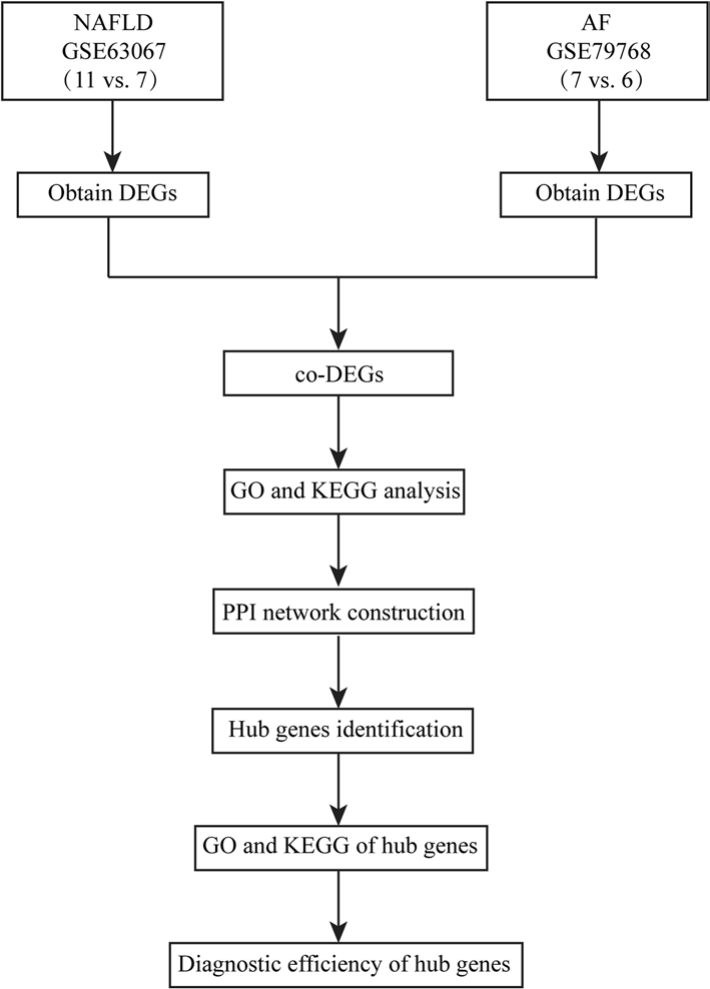
Flow diagram of the study design. NAFLD, nonalcoholic fatty liver disease; AF, atrial fibrillation; DEGs, differentially expressed genes; co-DEGs, co-expressed differentially expressed genes; GO, Gene Ontology; KEGG, Kyoto Encyclopedia of Genes and Genomes; PPI, protein–protein interaction

The NAFLD dataset (GSE63067) and AF dataset (GSE79768) were downloaded from GEO database. Both datasets were derived from the GPL570 platform (Affymetrix Human Genome U133 Plus 2.0 Array). The GSE63067 dataset contained the gene expression profiles of 11 NAFLD patients and 7 non-NAFLD controls. GSE79768 consisted of 13 pairs of left and right arterial appendages that were derived from 6 patients with sinus rhythm and 7 patients with persistent AF. Additional approval form the Ethics Committee was not required because the gene expression profiles included in the current study were downloaded from a free open-access database. The overall analysis for this study is shown in Fig. 1.

### Identification of DEGs in NAFLD and AF

The identification of DEGs between patients suffering from NAFLD or AF and healthy controls was performed using the R-platform (http://Rproject.org) and LIMMA package [23]. A |log_2_FC| larger than 0.5 was considered the threshold for differential expression, and a significant difference was defined as P value < 0.05. Genes with log_2_FC > 0 were considered upregulated genes, while those with log_2_FC < 0 were considered downregulated genes. The NAFLD-related (NAFLD-DEGs) and AF-related DEGs (AF-DEGs) were visualized in volcano plots and heatmaps via the R platform. To visualize the co-DEGs between NAFLD and persistent AF patients, an online tool (*bioinformatics.psb.ugent.be/webtools/Venn*) was used to draw a Venn diagram. These co-DEGs were used for subsequent analysis.

### Functional enrichment analysis of co-DEGs

To obtain the potential biological function and signaling pathways of the co-DEGs, the R package clusterProfiler [24] was applied to conduct GO analysis and KEGG pathway enrichment analysis. An adjusted P value < 0.05 was regarded as significant. Bubble diagrams and bar charts were constructed for the visualization of the results of the GO terms and KEGG pathway enrichment analyses, respectively. Similarly, we also performed a functional enrichment analysis of co-DEGs via Metascape (https://metascape.org) [25], which is a powerful online annotation analysis program that consists of GO terms such as cellular component (CC), molecular function (MF), biological process (BP), and KEGG pathway analysis.

### Construction of the PPI network of the co-DEGs

The PPI network of the co-DEGs was analyzed using the search tool for the retrieval of interacting genes (STRING database, Version 11.0; http://string-db.org/) that provides the predicted and experimental interactions of proteins, and a confidence score > 0.4 was defined as the cut-off value [26]. Subsequently, the PPI network was visualized with Cytoscape software (Version 3.8.0; http://cytoscape.org/). Furthermore, the plugin CytoHubba and the MCC method were used to calculate the protein score and obtain the top 10 hub genes with the highest scores [27].

### Correlation analysis between the hub genes and NAFLD/AF

The interconnectivity among genes related to NAFLD and AF was shown in the mapping of the Circos plot, which was constructed using R language. Using SPSS 23.0 (SPSS, Inc, Chicago, IL, USA), we performed binomial logistic regression analysis to examine correlations between hub gene expression and AF. The R package ggplot2 was used to draw a generalized linear model fitting cure.

### Enrichment analysis, expression analysis, and diagnostic analysis of the hub genes

The GO and KEGG enrichment analyses for the hub genes were completed via the R package clusterProfiler. The enrichment results were displayed by bubble diagrams and bar diagrams, which were drawn by R language. Two heatmaps of the hub gene expression levels were processed in Morpheus (https://software.broadinstitute.org/morpheus). Finally, we generated receiver operator characteristic (ROC) curves using the pROC R package and calculated the area under the curve (AUC) of the hub genes to determine the usefulness of these hub genes in predicting AF in NAFLD patients. A value of P < 0.05 was considered statistically significant.

### Identification of hub genes associated with liver or cardiovascular diseases

The Comparative Toxicogenomics Database (CTD; http://ctdbase.org/) [28] was used to find the integrated chemical-disease, chemical-gene, and gene-disease interactions to generate expanded networks and predict novel associations. Through these data, we analyzed the relationships between gene products and liver or cardiovascular diseases. Here, relationships between hub genes and diseases were identified.

### Cell culture and treatment

The human hepatocyte cell line Huh-7 was obtained from the American Type Culture Collection (ATCC, US) and cultured in a humidified incubator at 37 °C with 5% CO_2_ using Dulbecco’s Modified Eagle Medium (DMEM) (Gibco, US), which was supplemented with 1% penicillin and streptomycin (Invitrogen, US) and 10% fetal bovine serum (FBS) (Gibco, US). To establish the in vitro NAFLD cell model, Huh-7 cells were cultured in the presence or absence of 1 mM free fatty acids (FFA, containing oleic acid and palmitic acid at a 2:1 volume ratio) for 24 h and then used for the RNA extraction and quantitative Real-Time PCR (qRT-PCR).

### RNA extraction and qRT-PCR

Total RNA was extracted from Huh-7 cells by TRIzol reagent (Takara, Japan). A total of 1000 ng of extracted RNA was used to perform reverse transcription using Evo M-MLV RT Premix for qPCR (Accurate Biotechnology, China). qRT-PCR was performed using SYBR Green Premix Pro Taq HS qPCR Kit (Accurate Biotechnology, China) with the specific primers. Expression of the PCR data were shown as 2^−ΔΔct^, and normalized to the internal control level (GAPDH). The primers used in qRT-PCR are listed in Additional file 8: Table S2.

### Statistical analysis

All data are presented as the mean ± standard deviation (SD). Student’s *t*-test was used to compare differences between two groups. Statistical significance was indicated when P < 0.05. All statistical analyses were performed using GraphPad Prism 9.0 software (San Diego, CA, USA).

## Results

### Identification of DEGs in GSE63067 and GSE79768

**Fig. 2:**
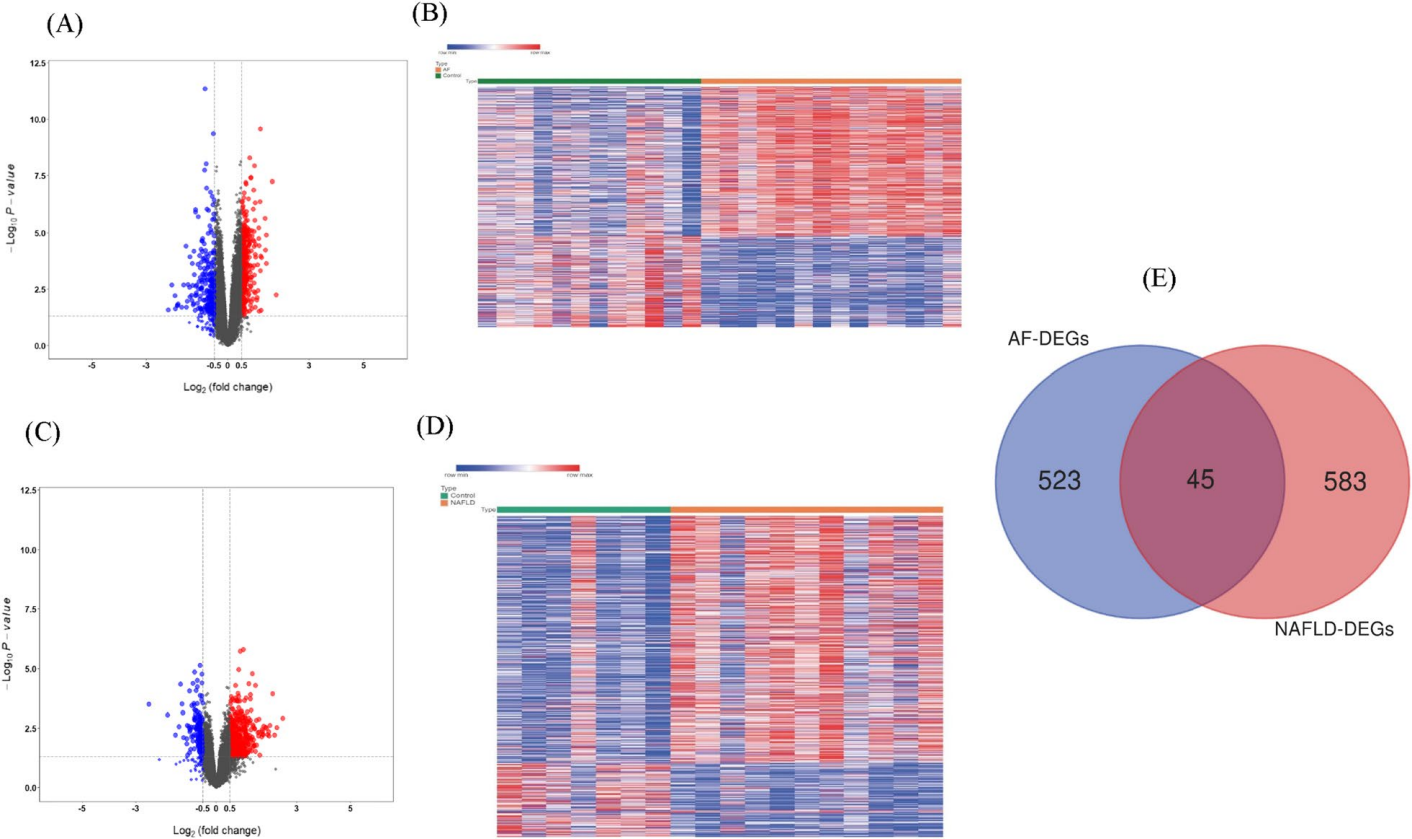
**A** Volcano plot of DEGs in AF. Blue represents downregulated DEGs, red represents upregulated DEGs, and black represents no difference. **B** Heatmap of the DEGs in AF patients compared with normal controls. **C** Volcano plot of DEGs in NAFLD. Blue represents downregulated DEGs, red represents upregulated DEGs, and black represents no difference. **D** Heatmap of the DEGs in NAFLD patients compared with normal controls. **E** Venn diagram of co-DEGs from the intersection of two independent datasets GSE79768 and GSE63067. DEGs, differentially expressed genes; AF, atrial fibrillation; NAFLD, nonalcoholic fatty liver disease

The basic information from the datasets related to NAFLD and AF is shown in Table 1. With P value < 0.05 and |log_2_FC|> 0.5 as the screening conditions, a total of 488 genes were upregulated and 140 genes were downregulated in NAFLD. In AF, a total of 353 genes were upregulated and 215 genes were downregulated. The results are shown in Additional file 1 and Additional file 2. Analysis of the volcano plots (Fig. 2A, C) and heatmap clustering (Fig. 2B, D) showed that the identified NAFLD-DEGs and AF-DEGs can easily distinguish patients with NAFLD or AF from healthy controls. The Venn diagram demonstrates that 45 DEGs were co-expressed in the two gene expression groups (Fig. 2E). The detailed information of the co-DEGs were shown in Additional file 7: Table S1.

**Table 1:** Details from the datasets related to AF or NAFLD patients

GEO ID	Disease type	Platform	Organism	Experiment type	Samples (case vs. control)	Country/region
GSE79768	AF	GPL570	*Homo sapiens*	Expression profiling by array	7 vs. 6	Taiwan, China
GSE63067	NAFLD	GPL570	*Homo sapiens*	Expression profiling by array	11 vs. 7	Sweden

AF, atrial fibrillation; NAFLD, nonalcoholic fatty liver disease; GEO, Gene Expression Omnibus

### Functional enrichment analyses of co-DEGs

**Fig. 3:**
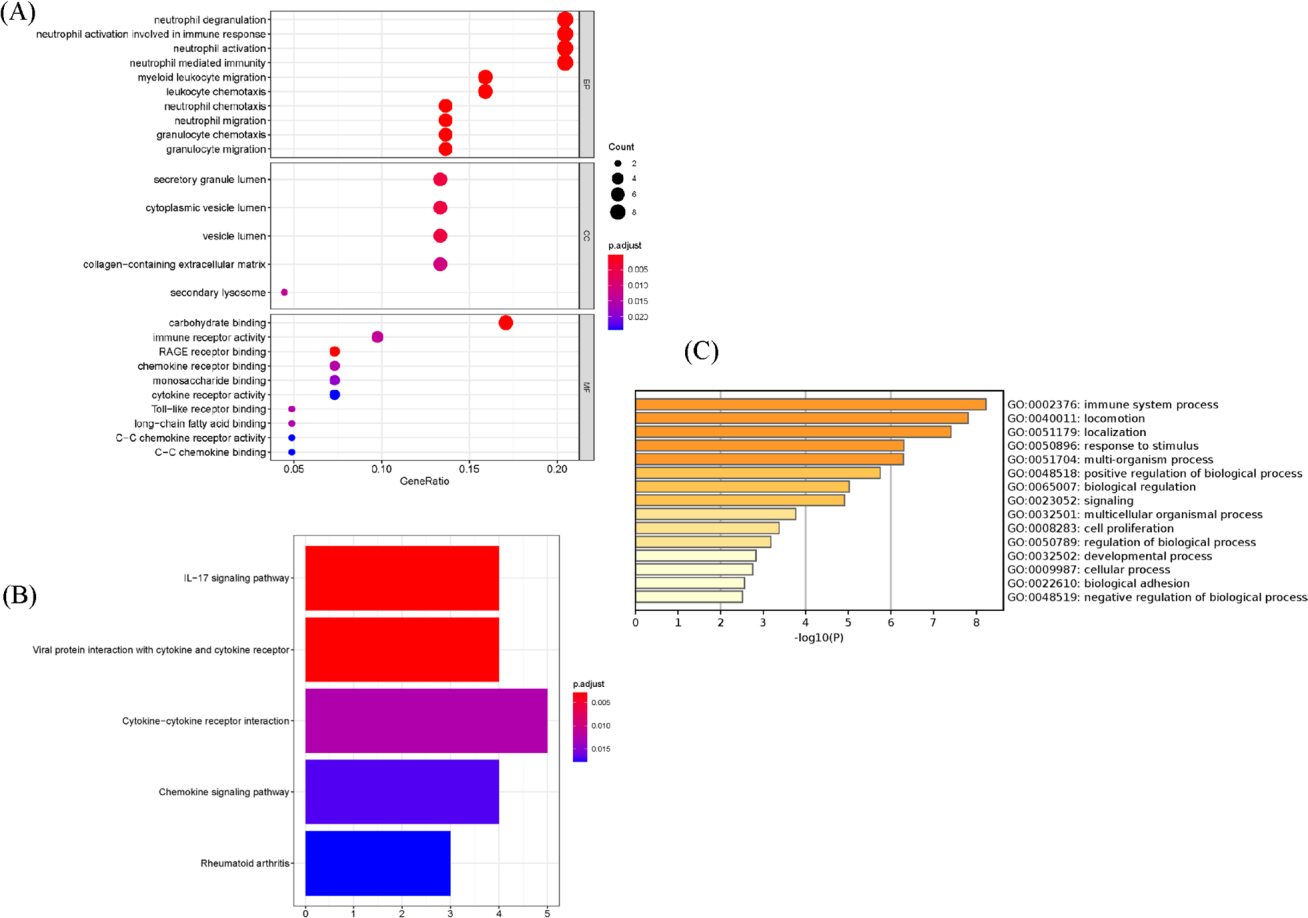
The enrichment analysis of co-DEGs by the R package clusterProfiler and Metascape. Detailed information relating to GO term enrichment in **A** BP, CC, and MF. **B** KEGG analysis for co-DEGs. **C** Heatmap of enriched terms across the co-DEGs via the online program Metascape

To gain more biological insight, GO enrichment analysis was performed by the clusterProfiler R package. The co-DEGs were classified into three functional groups: BP, CC, and MF. The GO enrichment analysis indicated that the changes in the BPs were primarily enriched in neutrophil degranulation, neutrophil activation involved in immune response, neutrophil activation, and neutrophil-mediated immunity (Fig. 3A and Additional file 3). Changes in the CCs for the co-DEGs were enriched mainly in the secretory granule lumen, cytoplasmic vesicle lumen, and vesicle lumen (Fig. 3A and Additional file 3). Changes in the MFs were significantly enriched in carbohydrate binding and immune receptor activity (Fig. 3A and Additional file 3). To investigate the crucial pathways of these co-DEGs, KEGG pathway analysis was performed, and the significant pathways are shown in Fig. 3B and Additional file 4. The co-DEGs were primarily enriched in the cytokine-cytokine receptor interaction, IL-17 signaling and chemokine signaling pathways. Additionally, functional enrichment analysis was also performed by Metascape and found that the co-DEGs were mainly enriched in immune system processes and locomotion (Fig. 3C).

### PPI network analysis

**Fig. 4:**
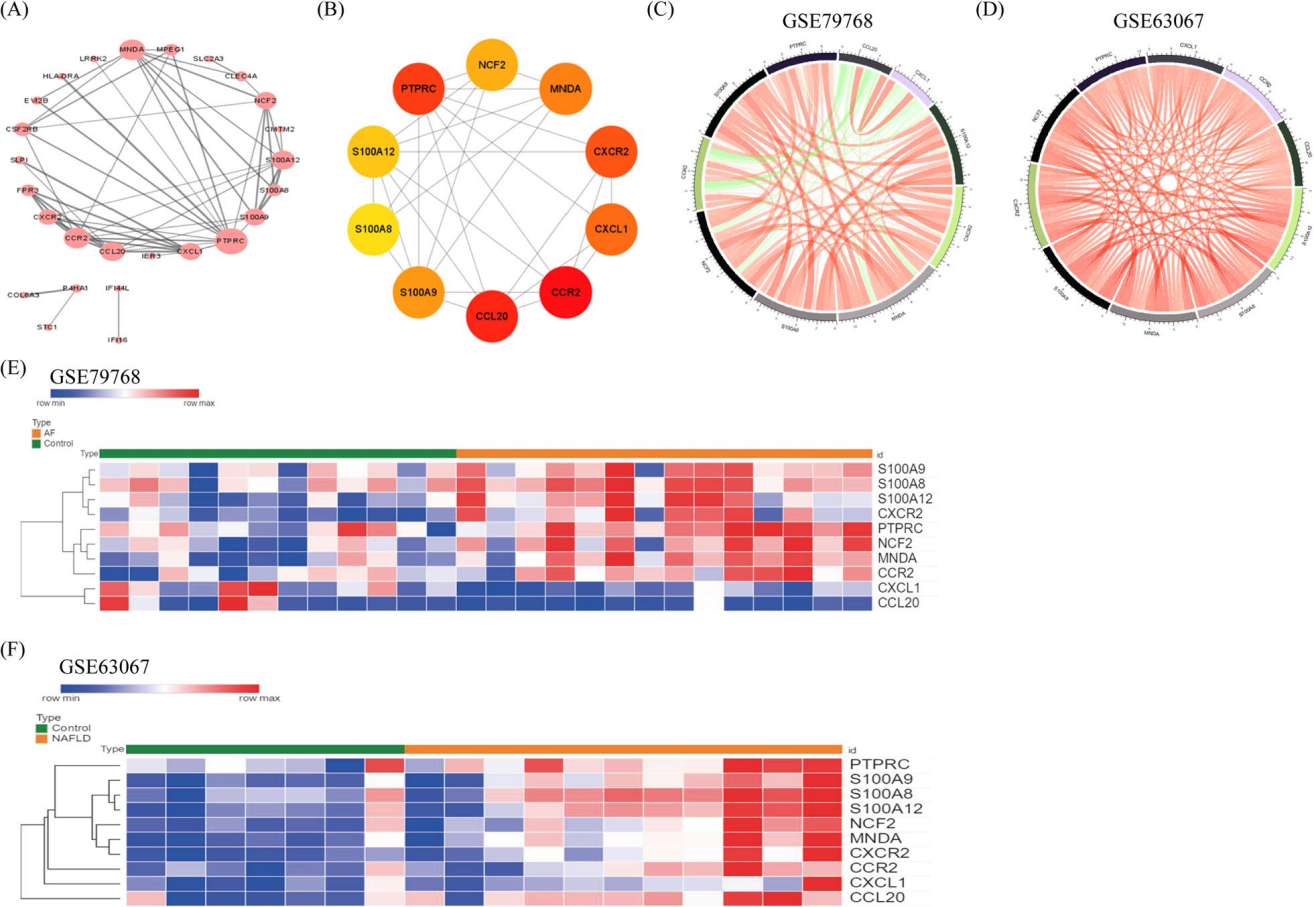
The PPI network of the co-DEGs, the hub gene network, the correlation analyses among the hub genes, and the expression profile analyses of the hub genes. **A** The PPI network includes 54 edges and 26 nodes. **B** The hub gene network (*S100A12*, *CXCR2*, *MNDA*, *S100A8*, *NCF2*, *CCR2*, *S100A9*, *PTPRC*, *CCL20*, and *CXCL1*). **C** The Circos plot showing that there are intense correlations among the hub genes in GSE79768. **D** Significant correlations among the hub genes were also performed in the GSE63067 dataset. **E** The expression profile of the hub genes for the control and AF samples in GSE79768. **F** The expression profile of the hub genes for the healthy group and NAFLD samples in GSE63067. Red indicates that gene expression is upregulated, and blue indicates that gene expression is downregulated. In the same color, the darker the color, the more significant it was

Using the STRING database, we performed PPI network analysis for the co-DEGs. We identified 54 edges and 26 nodes from the network of co-DEGs, and the data are shown in Fig. 4A. Furthermore, ten hub genes, including *CCR2*, *CCL20*, *PTPRC*, *CXCR2*, *CXCL1*, *MNDA*, *S100A9*, *NCF2*, *S100A12*, and *S100A8,* were identified from the PPI network owing to have the highest scores (Fig. 4B and Table 2).

**Table 2:** Hub genes with the highest scores

	Hub genes	Gene name	Score
AF	Upregulated genes	*CCR2*	72
		*PTPRC*	58
		*CXCR2*	54
		*MNDA*	46
		*S100A9*	42
		*NCF2*	38
		*S100A12*	37
		*S100A8*	24
	Downregulated genes	*CCL20*	68
		*CXCL1*	51
NAFLD	Upregulated genes	*CCR2*	72
		*CCL20*	68
		*PTPRC*	58
		*CXCR2*	54
		*CXCL1*	51
		*MNDA*	46
		*S100A9*	42
		*NCF2*	38
		*S100A12*	37
		*S100A8*	24

### Strong correlation between the hub genes and AF/NAFLD

**Fig. 5:**
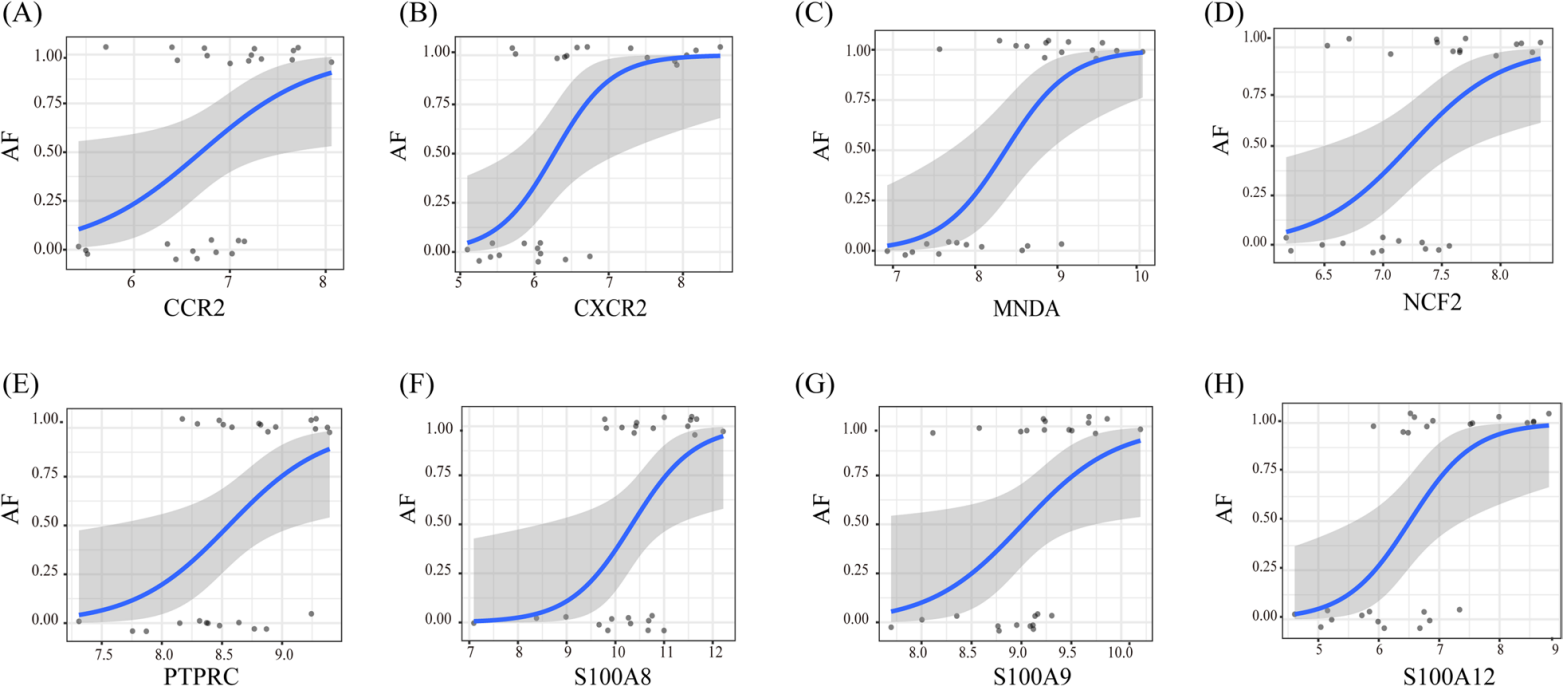
The relationship between hub gene expression and AF using the method of binomial logistic regression for generalized linear models. **A** *CCR2*, **B** *CXCR2*, **C** *MNDA*, **D** *NCF2*,** E** *PTPRC*, **F** *S100A8*, **G** *S100A9*, and **H** *S100A12*

By analyzing the expression profiles of ten hub genes in the GSE79768 dataset, a strong correlation between all the hub genes and AF was found, as shown in Fig. 4C. To further illustrate the differential expression of key genes in AF patients, we drew a heatmap that shows the expression data of the hub genes involved in the GSE79768 dataset (Fig. 4E). The expression of *CXCR2*, *PTPRC*, *CCR2*, *MNDA*, *NCF2*, *S100A9*, *S100A8*, and *S100A12* was upregulated in AF patients compared with the individuals in the control group. In addition, significant correlations were also confirmed in the GSE63067 dataset (Fig. 4D). Similarly, the expression levels of the hub genes in GSE63067 are shown in another heatmap (Fig. 4F). When compared with the individuals in the healthy group, the expression of *CXCR2*, *PTPRC*, *CCR2*, *MNDA*, *NCF2*, *S100A9*, *S100A8*, and *S100A12* was also upregulated in NAFLD patients. Univariate logistic regression analysis results suggested that *CCR2*, *PTPRC*, *CXCR2*, *MNDA S100A9*, *NCF2*, *S100A12,* and *S100A8* were significantly correlated with AF (Table 3). The shape of the relationship between hub gene expression and AF was then investigated through binomial logistic regression for generalized linear models. The results of this model indicated that the relationship was monotonic. From another perspective, the risk of AF increases with increased hub gene expression (Fig. 5).

**Table 3.** The logistic regression analyses between AF and relevant hub gene expression

Gene symbol	Coefficient	*P* value
*CCR2*	1.679	0.038
*PTPRC*	2.519	0.026
*CXCR2*	2.591	0.017
*MNDA*	2.557	0.006
*S100A9*	2.16	0.039
*NCF2*	2.534	0.014
*S100A12*	1.897	0.015
*S100A8*	1.57	0.028

Coefficient: regression coefficient (> 0, positive correlation; < 0, negative correlation)

### Functional GO terms and pathway enrichment analyses of the hub genes

**Fig. 6:**
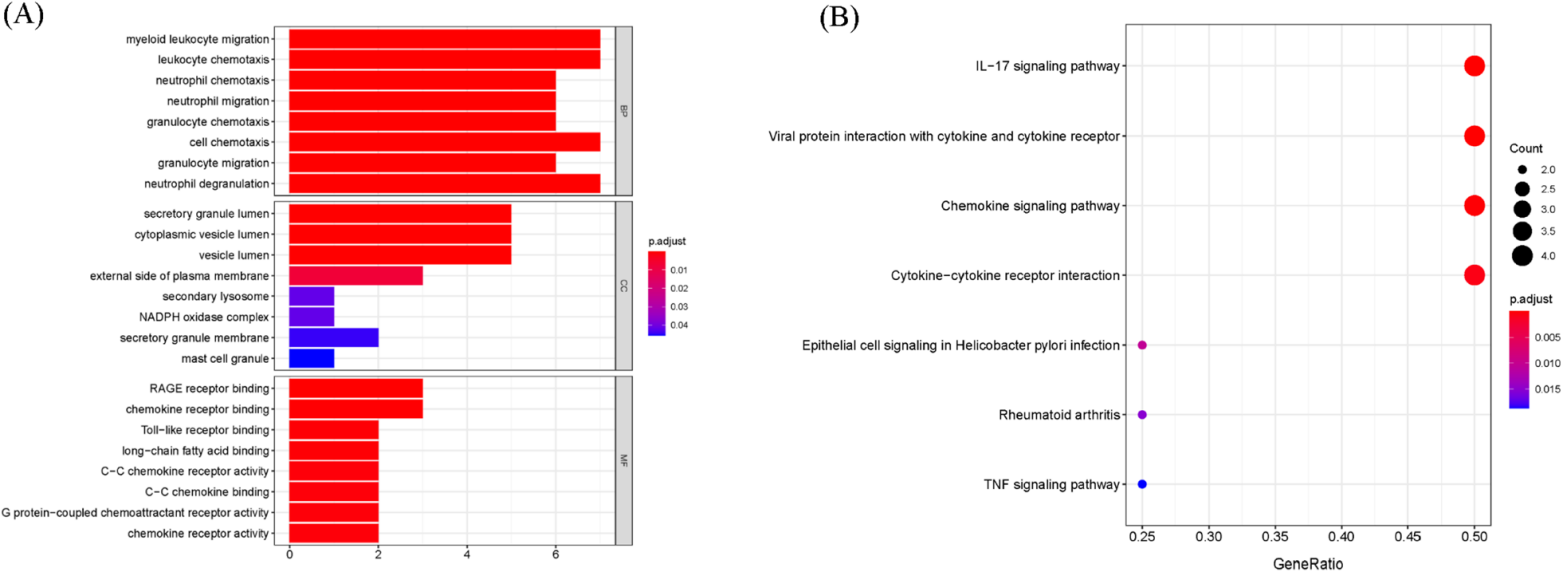
The enrichment analysis results of the hub genes. Specific information related to GO enrichment for the hub genes, including **A** BP, CC, and MF. **B** KEGG analysis for the hub genes

Functional enrichment analyses were conducted to reveal the functions of the hub genes, and the results are shown in Fig. 6. GO enrichment analysis revealed that the hub gene-related BPs were markedly concentrated in myeloid leukocyte migration, leukocyte chemotaxis, and neutrophil chemotaxis (Fig. 6A and Additional file 5). Regarding the CCs, the hub genes were significantly enriched in the secretory granule lumen, cytoplasmic vesicle lumen, and vesicle lumen (Fig. 6A and Additional file 5). In addition, MF analysis suggested that the hub genes were mainly involved in RAGE receptor binding, chemokine receptor binding, and Toll-like receptor binding (Fig. 6A and Additional file 5). To investigate the important pathways of these hub genes, KEGG pathway analysis was performed, and the significant pathways are shown in Fig. 6B and Additional file 6. The hub genes were significantly enriched in the IL-17 signaling pathway, viral protein interactions with cytokine and cytokine receptors, and the chemokine signaling pathway.

### Verification of the diagnostic value of the hub genes

**Fig. 7:**
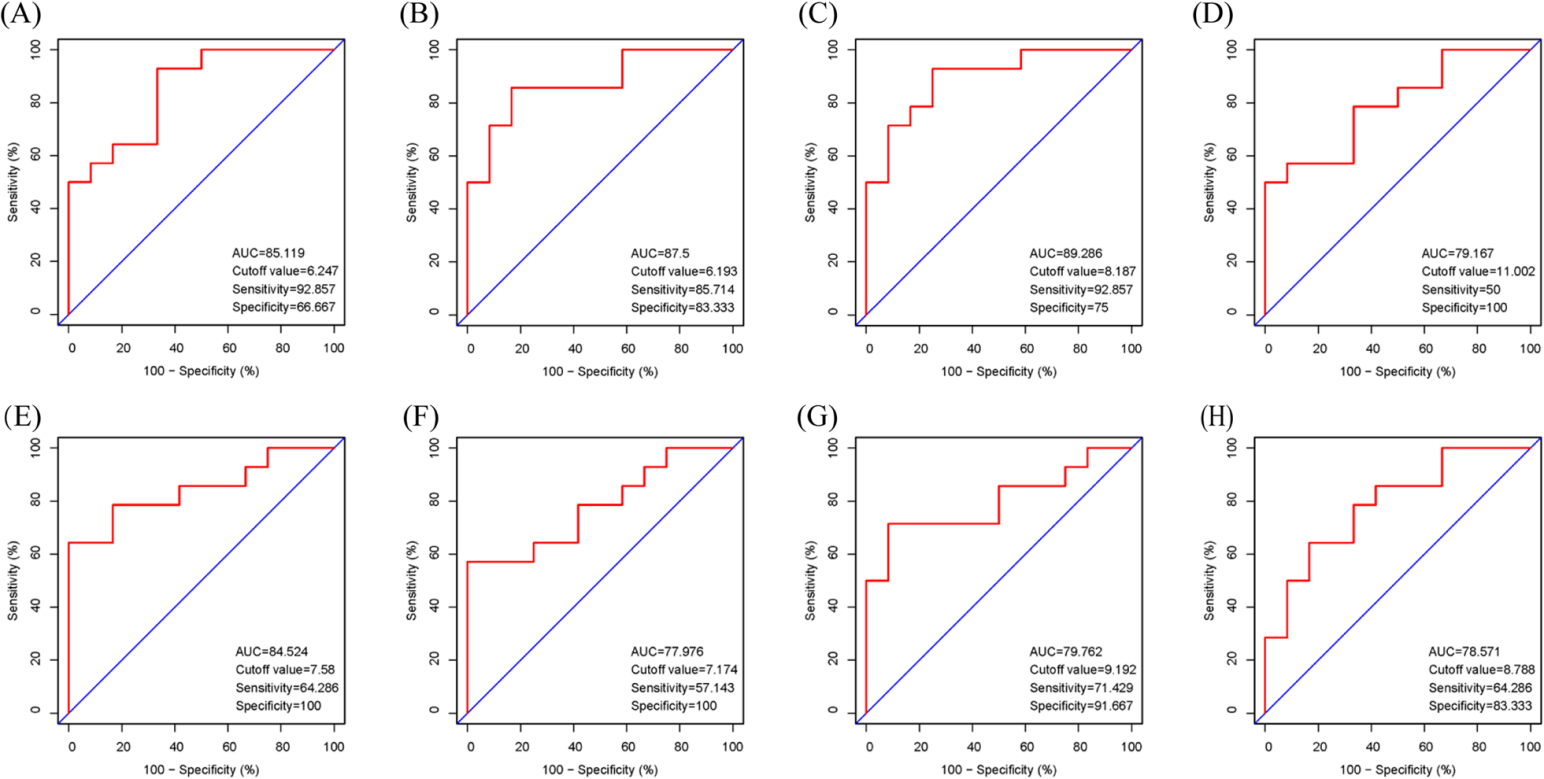
The ROC curves of the hub genes for AF. **A** *S100A12*, **B** *CXCR2*, **C** *MNDA*, **D** *S100A8*, **E** *NCF2*, **F** *CCR2*, **G** *S100A9*, and **H** *PTPRC*

To verify the diagnostic value of the hub genes acquired from the above analysis, we constructed ROC curves and calculated the corresponding AUC of these gene expression levels in the AF datasets. Figure 7 shows that *CXCR2*, *PTPRC*, *CCR2*, *MNDA*, *NCF2*, *S100A9*, *S100A8*, and *S100A12* were notably associated with the diagnosis of AF (70 < AUC < 100,  P < 0.05) (Table 4).

**Table 4:** Receiver operator characteristic curve analysis of the hub gene expression for AF

Gene symbol	AF		
	AUC	95% CI	Cutoff value
*S100A12*	85.119	70.471–99.767	6.247
*CXCR2*	87.5	73.89–100	6.193
*MNDA*	89.286	77.027–100	8.187
*S100A8*	79.167	61.728–96.605	11.002
*NCF2*	84.524	69.028–100	7.58
*CCR2*	77.976	59.897–96.056	7.174
*S100A9*	79.762	61.817–97.707	9.192
*PTPRC*	78.571	60.742–96.401	8.788

AUC, area under the curve; AF, atrial fibrillation

### Identification of the hub genes

**Fig. 8:**
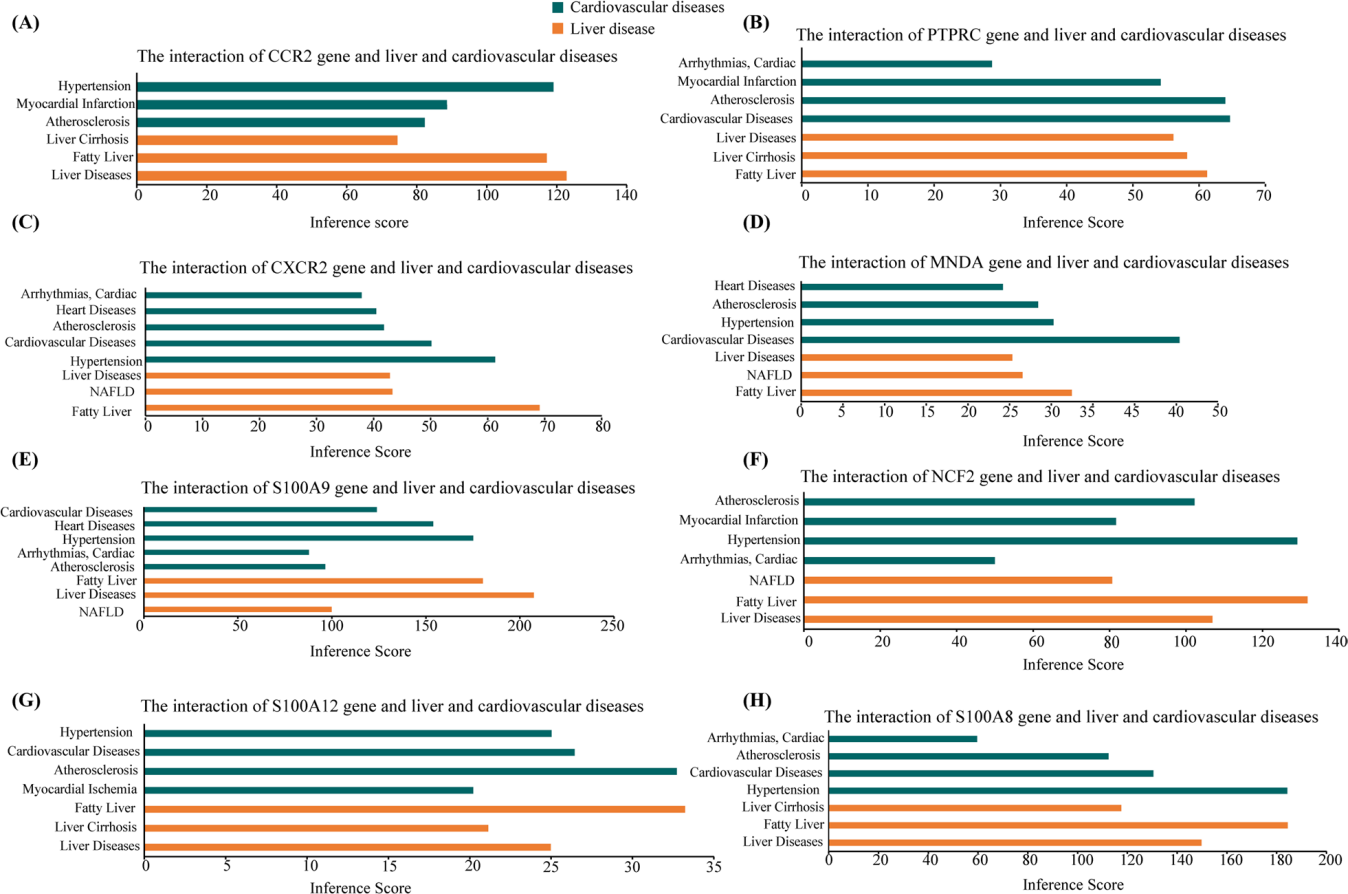
Relationships between the hub genes and liver or cardiovascular diseases identified in the CTD database. **A** *CCR2*, **B** *PTPRC*, **C** *CXCR2*, **D** *MNDA*, **E** *S100A9*, **F** *NCF2*, **G** *S100A12*, and **H** *S100A8.* NAFLD, nonalcoholic fatty liver disease

The CTD database demonstrated that the hub genes (*CXCR2*, *PTPRC*, *CCR2*, *MNDA*, *NCF2*, *S100A9*, *S100A8*, and *S100A12*) target several liver and cardiovascular diseases, and the results are displayed in Fig. 8.

### Validation of the hub genes in NAFLD cell model

**Fig. 9:**
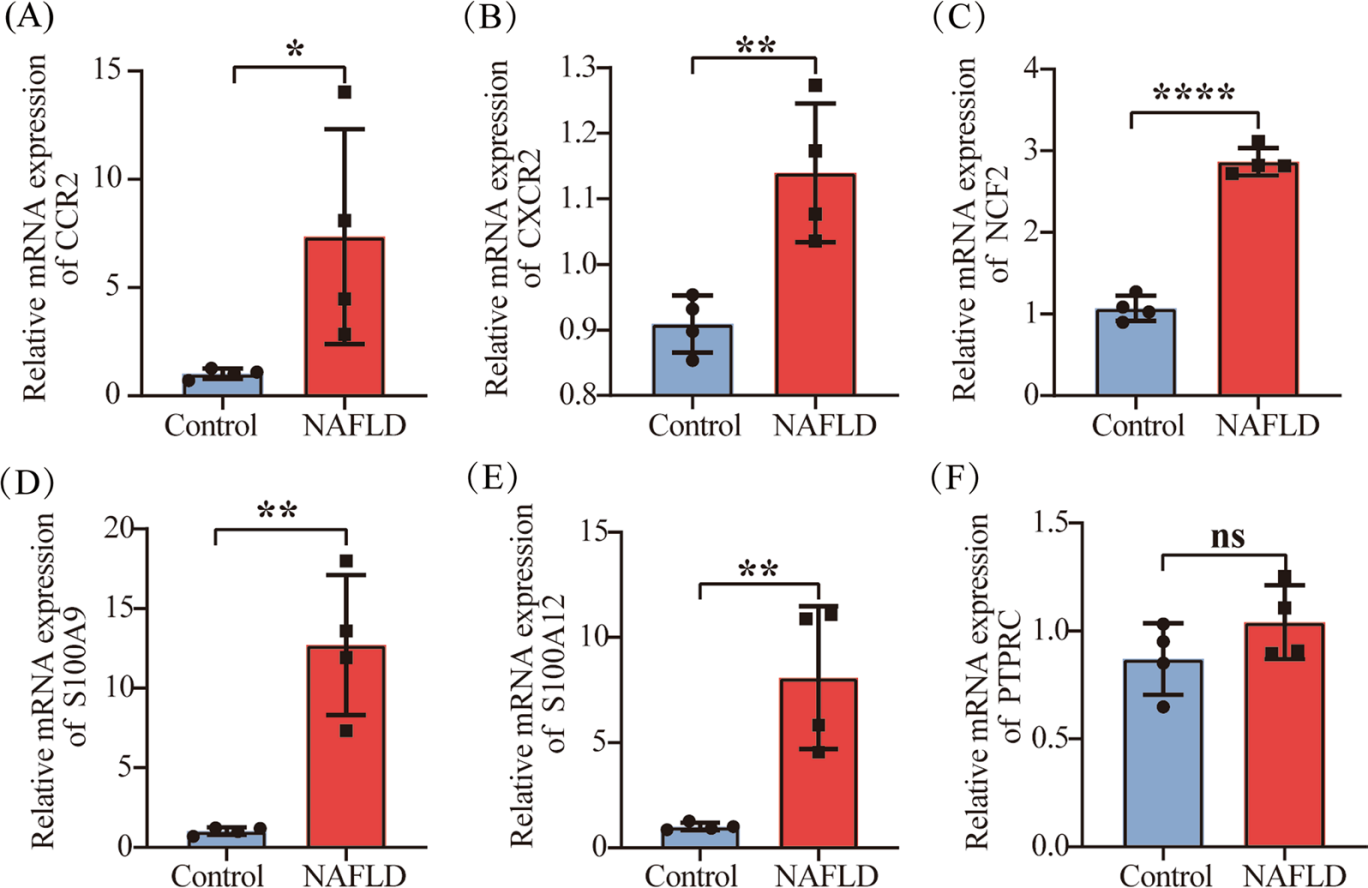
The mRNA expression levels of 8 hub genes were measured in NAFLD cell model and control group. **A** *CCR2*, **B** *CXCR2*, **C** *NCF2*, **D** *S100A9*, **E** *S100A12*, **F** *PTPRC*. * P < 0.05; ** P < 0.01; **** P < 0.0001. Abbreviation: ns, non-significant

To confirm the reliability of the bioinformatics analysis, we validated hub genes in NAFLD cell model by qRT-PCR. To mimic the NAFLD phenotype, Huh-7 cells were treated with FFA for 24 h. Similar to the results of mRNA microarray in NAFLD tissue samples, the expression levels of *CCR2*, *CXCR2*, *NCF2*, *S100A9*, *S100A12* were found to be significantly upregulated in cells treated with FFA (Fig. 9A–E). However, the expression level of *PTPRC* showed no significant difference between NAFLD cell model and control group, which did not correlate with our bioinformatics analysis (Fig. 9F). *S100A8* and *MNDA* were not detected by qRT-PCR due to their low expression levels.

## Discussion

AF is the most prevalent form of cardiac arrhythmia, and the prevalence and mortality of AF are still increasing globally [29]. Although many risk factors, such as age, hypertension, and diabetes, have been proposed [29], the incidence and prevalence are still high. Therefore, new causes need to be clarified. NAFLD has been identified as the most common liver and metabolic disease worldwide, with a high global prevalence [30]. Increasing evidence has reported that NAFLD is a multisystem disease [31]. Mounting evidence has indicated a strong relationship between NAFLD and cardiovascular disease [32]. However, NAFLD has not been fully deemed an important risk factor by cardiologists, and NAFLD has not been included in the guidelines as a main risk factor for the prevention and treatment of cardiovascular diseases [30]. Interestingly, many clinical studies have suggested that NAFLD patients have a higher prevalence of arrhythmias such as AF [10, 33]. However, the potential pathophysiological mechanisms of NAFLD and AF are quite complex. To elucidate the molecular mechanism in these two diseases, new molecules need to be revealed. Thus, in the current study, an effective method of bioinformatics technology was applied to mine valuable data and analyze complicated genetic networks.

We reviewed the previous literature and found that several putative mechanisms potentially link NAFLD and AF. Certain changes occur in the liver of NAFLD patients that could lead to disturbances in the liver-heart crosstalk networks. First, NAFLD might lead to increased systematic inflammation, which might trigger AF [34]. Second, the increased thickness of epicardial fat and dysregulation of glucose and lipid metabolism in NAFLD patients has been shown to be linked to the dysfunction of left ventricular diastolic, which is a potential hazard for AF [35]. The thickened epicardial fat acts as an endocrine organ and secretes proinflammatory cytokines and prooxidant molecules, which might result in morphological and functional cardiac alterations [36]. Finally, NAFLD has also been indicated to be related to the disturbance of immunologic homeostasis, which may play crucial roles in AF initiation and progression [37]. These NAFLD-associated changes might play important roles in the structural, electrical, and autonomic remodeling of the left atrium [38]. Therefore, the inflammatory response and immune dysfunction are very important in the progression of NAFLD and AF. The link between the two diseases could have crucial clinical implications for patients with NAFLD and emphasize prophylaxis against AF.

In the current study, 45 co-DEGs were identified between NAFLD/AF and healthy controls based on large mRNA expression datasets. These co-DEGs were primarily linked to immune and inflammatory responses, such as neutrophil degranulation, neutrophil activation involved in immune response, and neutrophil activation, and significantly enriched in inflammation pathways, such as cytokine-cytokine receptor interaction, IL-17 signaling pathway, and chemokine signaling pathway. PPI network analyses showed that the hub genes (*CCR2*, *PTPRC*, *CXCR2*, *MNDA*, *S100A9*, *NCF2*, *S100A12*, and *S100A8*) were upregulated in both NAFLD and AF datasets and may play crucial roles in the pathogenesis of NAFLD and AF. These genes were also significantly enriched in immune- and inflammatory- related responses, including leukocyte chemotaxis, neutrophil chemotaxis, the IL-17 signaling pathway, viral protein interactions with cytokines and cytokine receptors, and the chemokine signaling pathway.

AF is reported to be associated with systemic inflammation [39]; simultaneously, systematic inflammation contributes to the progression of NAFLD [9]. The inflammatory and immune responses are mainly associated with AF and NAFLD occurrence. The expanding white adipose tissue (WAT) in obesity may constitute an important origin of inflammation during the development of NAFLD. As a chemokine receptor, C–C chemokine receptor type 2 (CCR2) regulates the immune response by inducing macrophage and monocyte recruitment to sites of inflammation and promoting inflammatory diseases [40]. The CCR2 level and chemotactic activity have been reported to be significantly elevated in obese subjects [41]. Additionally, CCR2 plays a crucial role in the recruitment of immune cells to WAT and the liver, promoting the inflammatory component of the disease. Mulder et al. indicated that inhibition of CCL2-CCR2 pathways may be a promising strategy to reduce the onset and progression of hepatic fat accumulation and inflammation in aged mice [42]. Through bioinformatics analysis, Gang Fan et al. found that the expression of CCR2 was elevated in AF patients [43]. Miyosawa et al. reported that the protein level of CCR2 was higher in isolated monocytes of AF patients whose left atrial diameter was larger [44]. Taken together, these reports are in agreement with our current demonstration that *CCR2*, as a hub gene, was overexpressed in both NAFLD and AF datasets. CCR2 might serve as a potential biomarker for predicting AF in NAFLD patients. AF is often related to an intense inflammatory response characterized by the infiltration of monocytes/macrophages. Chemokine (C-X-C motif) receptor 2 (CXCR2) plays a critical role in promoting the recruitment of neutrophils and monocytes/macrophages into the injured heart and arterial wall [45]. Zhang et al. elucidated that CXCR2 plays an important role in propelling monocyte infiltration towards the atria and accelerates atrial remodeling and AF occurrence. In addition, they also reported an increased number of CXCR2^+^ monocyte counts in AF patients [46]. Through experiments, Zhang et al. further confirmed that the inhibition of CXCR2 prevented and reversed hypertension-induced AF and atrial remodeling, providing evidence that CXCR2 might be a potential therapeutic target for AF [47]. Liver inflammatory cell infiltration is also a characteristic of NAFLD. Ye et al. demonstrated that both the expression of neutrophil-derived lipocalin 2 (LCN2) and the chemokine CXCR2 were significantly elevated in mouse models and human patients with NAFLD. They also found that, LCN2-CXCR2 gave rise to the activation of the mitogen-activated protein (MAP) kinase ERK1/2 and the generation of chemokines that induce the infiltration of inflammatory cells to regulate the pathogenesis of NAFLD [48]. Monocyte-derived macrophages have a crucial role in the progression of NAFLD. Additionally, the CCL2-CCR2 axis, which is primarily produced by monocytes or macrophages, can also regulate monocyte flux to liver tissue [49]. In addition, our GO enrichment analysis results indicated that *S100A12*, *CXCR2*, *S100A8*, *CCR2*, *S100A9*, *CXCL1*, and *CCL20* may be involved in myeloid leukocyte migration. Therefore, CXCR2 and CCR2 might be significantly associated with both NAFLD and AF patients and might be potential biomarkers of NAFLD-related AF. A high density of myeloid and lymphoid immune cells plays important roles in the development and progression of NAFLD [50]. *S100A8* has been shown to play an important role as an endogenous immune-activator in inflammatory diseases. Mukai et al. found that the expression of *S100A8* was significantly elevated in both a diet model of NAFLD and in NAFLD patients, and it also induced the production of TNFα and the development of NAFLD [51]. Mounting studies have indicated that proinflammatory mediators might lead to myocardial structural and functional remodeling [52]. A basic research study led by Aschar-Sobbi R suggested that high levels of TNFα were associated with atrial remodeling and could be mitigated by *Tnf* gene ablation [53].

Through microarray technology, Liu et al. also found that the gene expression of *S100A9* was higher in NAFLD rat livers. The serum level of S100A9 was correlated with the NAFLD Activity Score and the severity of hepatic steatosis [54]. Interestingly, through single-cell RNA sequencing analysis, downregulated *S100A8/A9* was found in macrophages and dendritic cells of the NAFLD progression process [55]. We found that the expression levels of *S100A8/A9/A12* were upregulated in both NAFLD and AF patients; however, the functions of these proteins in AF progression still need further investigations.

The novel variants of neutrophil cytosolic factor 2 (NCF2) are rate-limiting cofactors of NADPH oxidase that are necessary for reactive oxygen species production in phagocytes and are used as markers for oxidative stress, which play important roles in innate immunity [56]. NCF2 is highly expressed in NAFLD and contribute to oxidative stress in NAFLD [57]. It should be noted that the relationship between NCF2 and AF has not been elucidated and needs further exploration.

In addition, study on MNDA, PTPRC and AF or NAFLD is not reported and needs more investigations.

To analyze the functions and pathways that are enriched for the hub genes, significant GO BP terms and pathways were obtained. In the present study, enrichment analysis of KEGG pathways indicated that the *S100A8* and *S100A9* genes may be involved in the Toll-like receptor signaling pathway. The Toll-like receptor signaling pathway plays an important role in adaptive and innate immune responses. Inflammation is always triggered by innate and/or adaptive immune responses. Emerging evidence suggests that innate immune signaling is an important factor promoting hepatic inflammation [58]. It is becoming increasingly clear that innate immune signaling, in a multitude of processes, modulates the progression of metabolic diseases such as NAFLD and cardiovascular diseases, which are characterized by low-grade inflammation and metabolic disequilibrium [59]. Thus, we speculated that these hub genes might exert their biological functions through the Toll-like receptor signaling pathway in both NAFLD and AF.

TNF is a multifunctional cytokine, and most cells show at least some TNF responsiveness. In general, TNF plays a vital role in innate immunity by promoting the expression and activation of several genes that are associated with inflammatory responses. Different immune cells, mainly macrophages and lymphocytes, can produce TNF [60]. Emerging evidence demonstrates that TNFα positively correlates with the progression of AF from paroxysmal to persistent forms and can predict the prognosis of AF ablation [61]. Increased levels of TNFα signaling might promote the remodeling of arterial electrical, structural, and contractile components, all of which are important components of the molecular pathophysiology of AF [62]. TNF signaling activation is also implicated in the activation of the NF-κB and p38-MAPK pathways in an AF model [53]. NF-κB is a transcription factor and can translocate to the nucleus to regulate the transcription of genes related to inflammatory and immune responses [63]. In addition, the activation of NF-κB also leads to modulation of ion channels and transcription factors involved in AF development [64]. Overall, TNF and NF-κB appear to serve as key factors in inflammatory signaling in the process of AF. Besides, accumulating evidence has also suggested that NF-κB plays a significant role in NAFLD progression. It is well known that the NF-κB signaling pathway can promote the transformation from liver tissue steatosis to steatohepatitis [65]. These results indicate that the TNF and NF-κB signaling pathways might participate in the development of NAFLD and AF.

In addition, several main BPs that may participate in NAFLD and AF were recognized by the GO functional enrichment analysis of the co-DEGs and hub genes. These included, the chemokine-mediated signaling pathway (GO:0070098) (*CXCR2*, *CCR2*), the positive regulation of inflammatory response (GO:0050729) (*S100A12*, *S100A8*, *CCR2*, and *S100A9*), mononuclear cell migration (GO:0071674) (*S100A12*, *CCR2*), the positive regulation of tumor necrosis factor superfamily cytokine production (GO:1903557) (*CCR2*, *PTPRC*), and the response to chemokines (GO:1990868) (*CXCR2*, *CCR2*). Thus, the combination of previous and current research results further revealed that *CCR2*, *CXCR2*, *S100A9*, *PTPRC*, *S100A8*, *S100A12* might be involved in inflammatory and immune processes that eventually result in NAFLD and AF. Hence, there may be a correlation between the liver and cardiovascular diseases.

### Limitations

Despite this study’s bioinformatics analysis, the results of this research should be interpreted within the context of crucial limitations. First, only two mRNA expression profiles were included in this study. The relatively small number of samples may make the results less convincing. Second, this study lacks further mechanistic validation. qRT-PCR was performed to verify the expression levels of *CXCR2*, *PTPRC*, *CCR2*, *MNDA*, *NCF2*, *S100A9*, *S100A8* and *S100A12*. The expression levels of *CXCR2*, *CCR2*, *NCF2*, *S100A9*, *S100A12* in NAFLD cell model were significantly higher than control group. Due to the short of AF samples, the obtained hub genes should be further verified by in vitro and in vivo in AF samples. Thereby laying a foundation for clarifying key molecular targets of NAFLD and AF.

## Conclusions

In summary, through the gene differential expression analysis, functional enrichment analysis, and PPI analysis of DEGs in AF and NAFLD we have successfully provided deeper insight to the molecular changes in AF and NAFLD pathogenesis, and identified several potential candidate therapeutic changes, including *CXCR2*, *PTPRC*, *CCR2*, *MNDA*, *NCF2*, *S100A9*, *S100A8*, *S100A12*. These hub genes were mainly enriched in inflammation and immune related biological functions and pathways. Our results may provide new clues for exploring the pathogenesis of AF and NAFLD from the perspective of genetics.

## Supplementary Information

**Additional file 1.** DEGs involved in NAFLD samples.

**Additional file 2.** DEGs involved in AF samples.

**Additional file 3.** GO terms enrichment analysis results of co-DEGs.

**Additional file 4.** KEGG pathway enrichment analysis results of co-DEGs.

**Additional file 5.** GO terms enrichment analysis results of hub genes.

**Additional file 6.** KEGG pathway enrichment analysis results of the hub genes.

**Additional file 7: Table S1.** Forty-five common genes of NAFLD and AF.

**Additional file 8: Table S2.** Primer Sequences for qRT-PCR.

## Data Availability

The datasets used and analyzed during the current study are available in the GEO database through GEO accession numbers GSE63067 and GSE79768.

## Abbreviations

AF: Atrial fibrillation
NAFLD: Nonalcoholic fatty liver disease
DEGs: Differentially expressed genes
GEO: Gene Expression Omnibus
GO: Gene Ontology
KEGG: Kyoto Encyclopedia of Genes and Genomes
PPI: Protein–protein interaction
co-DEGs: Co-expressed differentially expressed genes
CC: Cellular component
MF: Molecular function
BP: Biological process
ROC: Receiver operator characteristic
CTD: Comparative Toxicogenomics Database
AUC: Area under the curve
FFA: Free fatty acids
ATCC: American Type Culture Collection
FBS: Fetal bovine serum
qRT-PCR: Quantitative Real-Time PCR
WAT: White adipose tissue

## References

[1] Kirchhof P, Benussi S, Kotecha D, Ahlsson A, Atar D, Casadei B, Castella M, Diener HC, Heidbuchel H, Hendriks J (2016). 2016 ESC Guidelines for the management of atrial fibrillation developed in collaboration with EACTS. Europace.

[2] Chugh SS, Havmoeller R, Narayanan K, Singh D, Rienstra M, Benjamin EJ, Gillum RF, Kim YH, McAnulty JH, Zheng ZJ (2014). Worldwide epidemiology of atrial fibrillation: a Global Burden of Disease 2010 Study. Circulation.

[3] Freeman JV, Wang Y, Akar J, Desai N, Krumholz H (2017). National trends in atrial fibrillation hospitalization, readmission, and mortality for medicare beneficiaries, 1999–2013. Circulation.

[4] Rahman F, Kwan GF, Benjamin EJ (2014). Global epidemiology of atrial fibrillation. Nat Rev Cardiol.

[5] Guo Y, Lip GY, Apostolakis S (2012). Inflammation in atrial fibrillation. J Am Coll Cardiol.

[6] Kim MH, Johnston SS, Chu BC, Dalal MR, Schulman KL (2011). Estimation of total incremental health care costs in patients with atrial fibrillation in the United States. Circ Cardiovasc Qual Outcomes.

[7] European Association for the Study of the Liver (EASL), European Association for the Study of Diabetes (EASD), European Association for the Study of Obesity (EASO) (2016). EASL-EASD-EASO Clinical Practice Guidelines for the management of non-alcoholic fatty liver disease. J Hepatol.

[8] Fazel Y, Koenig AB, Sayiner M, Goodman ZD, Younossi ZM (2016). Epidemiology and natural history of non-alcoholic fatty liver disease. Metabolism.

[9] Buzzetti E, Pinzani M, Tsochatzis EA (2016). The multiple-hit pathogenesis of non-alcoholic fatty liver disease (NAFLD). Metabolism.

[10] Anstee QM, Mantovani A, Tilg H, Targher G (2018). Risk of cardiomyopathy and cardiac arrhythmias in patients with nonalcoholic fatty liver disease. Nat Rev Gastroenterol Hepatol.

[11] Sinner MF, Wang N, Fox CS, Fontes JD, Rienstra M, Magnani JW, Vasan RS, Calderwood AH, Pencina M, Sullivan LM (2013). Relation of circulating liver transaminase concentrations to risk of new-onset atrial fibrillation. Am J Cardiol.

[12] Targher G, Mantovani A, Pichiri I, Rigolon R, Dauriz M, Zoppini G, Morani G, Vassanelli C, Bonora E (2013). Non-alcoholic fatty liver disease is associated with an increased prevalence of atrial fibrillation in hospitalized patients with type 2 diabetes. Clin Sci (Lond).

[13] Byrne CD, Targher G (2015). NAFLD: a multisystem disease. J Hepatol.

[14] Karajamaki AJ, Hukkanen J, Ukkola O (2018). The association of non-alcoholic fatty liver disease and atrial fibrillation: a review. Ann Med.

[15] Polyzos SA, Mantzoros CS (2016). Adiponectin as a target for the treatment of nonalcoholic steatohepatitis with thiazolidinediones: A systematic review. Metabolism.

[16] Wang O, Chin R, Cheng X, Wu MKY, Mao Q, Tang J, Sun Y, Anderson E, Lam HK, Chen D (2019). Efficient and unique cobarcoding of second-generation sequencing reads from long DNA molecules enabling cost-effective and accurate sequencing, haplotyping, and de novo assembly. Genome Res.

[17] Zou R, Zhang D, Lv L, Shi W, Song Z, Yi B, Lai B, Chen Q, Yang S, Hua P (2019). Bioinformatic gene analysis for potential biomarkers and therapeutic targets of atrial fibrillation-related stroke. J Transl Med.

[18] Jia X, Zhai T (2019). Integrated Analysis of Multiple Microarray Studies to Identify Novel Gene Signatures in Non-alcoholic Fatty Liver Disease. Front Endocrinol (Lausanne).

[19] Zhu Y, Yang T, Duan J, Mu N, Zhang T (2019). MALAT1/miR-15b-5p/MAPK1 mediates endothelial progenitor cells autophagy and affects coronary atherosclerotic heart disease via mTOR signaling pathway. Aging (Albany NY).

[20] Zhang YF, Meng LB, Hao ML, Yang JF, Zou T (2020). Identification of co-expressed genes between atrial fibrillation and stroke. Front Neurol.

[21] Li W, Wang L, Wu Y, Yuan Z, Zhou J (2020). Weighted gene coexpression network analysis to identify key modules and hub genes associated with atrial fibrillation. Int J Mol Med.

[22] Barrett T, Wilhite SE, Ledoux P, Evangelista C, Kim IF, Tomashevsky M, Marshall KA, Phillippy KH, Sherman PM, Holko M (2013). NCBI GEO: archive for functional genomics data sets–update. Nucleic Acids Res.

[23] Ritchie ME, Phipson B, Wu D, Hu Y, Law CW, Shi W, Smyth GK (2015). limma powers differential expression analyses for RNA-sequencing and microarray studies. Nucleic Acids Res.

[24] Yu G, Wang LG, Han Y, He QY (2012). clusterProfiler: an R package for comparing biological themes among gene clusters. OMICS.

[25] Zhou Y, Zhou B, Pache L, Chang M, Khodabakhshi AH, Tanaseichuk O, Benner C, Chanda SK (2019). Metascape provides a biologist-oriented resource for the analysis of systems-level datasets. Nat Commun.

[26] Szklarczyk D, Gable AL, Lyon D, Junge A, Wyder S, Huerta-Cepas J, Simonovic M, Doncheva NT, Morris JH, Bork P (2019). STRING v11: protein-protein association networks with increased coverage, supporting functional discovery in genome-wide experimental datasets. Nucleic Acids Res.

[27] Shannon P, Markiel A, Ozier O, Baliga NS, Wang JT, Ramage D, Amin N, Schwikowski B, Ideker T (2003). Cytoscape: a software environment for integrated models of biomolecular interaction networks. Genome Res.

[28] Davis AP, Grondin CJ, Johnson RJ, Sciaky D, King BL, McMorran R, Wiegers J, Wiegers TC, Mattingly CJ (2017). The Comparative Toxicogenomics Database: update 2017. Nucleic Acids Res.

[29] Kornej J, Borschel CS, Benjamin EJ, Schnabel RB (2020). Epidemiology of atrial fibrillation in the 21st century: novel methods and new insights. Circ Res.

[30] Cai J, Zhang XJ, Li H (2019). Progress and challenges in the prevention and control of nonalcoholic fatty liver disease. Med Res Rev.

[31] Zhao YC, Zhao GJ, Chen Z, She ZG, Cai J, Li H (2020). Nonalcoholic fatty liver disease: an emerging driver of hypertension. Hypertension.

[32] Targher G, Byrne CD, Tilg H (2020). NAFLD and increased risk of cardiovascular disease: clinical associations, pathophysiological mechanisms and pharmacological implications. Gut.

[33] Stahl EP, Dhindsa DS, Lee SK, Sandesara PB, Chalasani NP, Sperling LS (2019). Nonalcoholic fatty liver disease and the heart: JACC state-of-the-art review. J Am Coll Cardiol.

[34] Ndumele CE, Nasir K, Conceicao RD, Carvalho JA, Blumenthal RS, Santos RD (2011). Hepatic steatosis, obesity, and the metabolic syndrome are independently and additively associated with increased systemic inflammation. Arterioscler Thromb Vasc Biol.

[35] Petta S, Argano C, Colomba D, Camma C, Di Marco V, Cabibi D, Tuttolomondo A, Marchesini G, Pinto A, Licata G, Craxi A (2015). Epicardial fat, cardiac geometry and cardiac function in patients with non-alcoholic fatty liver disease: association with the severity of liver disease. J Hepatol.

[36] Iacobellis G, Bianco AC (2011). Epicardial adipose tissue: emerging physiological, pathophysiological and clinical features. Trends Endocrinol Metab.

[37] Liu YC, Hung CS, Wu YW, Lee YC, Lin YH, Lin C, Lo MT, Chan CC, Ma HP, Ho YL, Chen CH (2013). Influence of non-alcoholic fatty liver disease on autonomic changes evaluated by the time domain, frequency domain, and symbolic dynamics of heart rate variability. PLoS ONE.

[38] Chen Z, Liu J, Zhou F, Li H, Zhang XJ, She ZG, Lu Z, Cai J, Li H (2021). Nonalcoholic fatty liver disease: an emerging driver of cardiac arrhythmia. Circ Res.

[39] Harada M, Van Wagoner DR, Nattel S (2015). Role of inflammation in atrial fibrillation pathophysiology and management. Circ J.

[40] Franca CN, Izar MCO, Hortencio MNS, do Amaral JB, Ferreira CES, Tuleta ID, Fonseca FAH (2017). Monocyte subtypes and the CCR2 chemokine receptor in cardiovascular disease. Clin Sci (Lond).

[41] Krinninger P, Ensenauer R, Ehlers K, Rauh K, Stoll J, Krauss-Etschmann S, Hauner H, Laumen H (2014). Peripheral monocytes of obese women display increased chemokine receptor expression and migration capacity. J Clin Endocrinol Metab.

[42] Stahl EC, Delgado ER, Alencastro F, LoPresti ST, Wilkinson PD, Roy N, Haschak MJ, Skillen CD, Monga SP, Duncan AW, Brown BN (2020). Inflammation and ectopic fat deposition in the aging murine liver is influenced by CCR2. Am J Pathol.

[43] Fan G, Wei J (2020). Identification of potential novel biomarkers and therapeutic targets involved in human atrial fibrillation based on bioinformatics analysis. Kardiol Pol.

[44] Miyosawa K, Iwata H, Minami-Takano A, Hayashi H, Tabuchi H, Sekita G, Kadoguchi T, Ishii K, Nozaki Y, Funamizu T (2020). Enhanced monocyte migratory activity in the pathogenesis of structural remodeling in atrial fibrillation. PLoS ONE.

[45] Abu Nabah YN, Losada M, Estelles R, Mateo T, Company C, Piqueras L, Lopez-Gines C, Sarau H, Cortijo J, Morcillo EJ (2007). CXCR2 blockade impairs angiotensin II-induced CC chemokine synthesis and mononuclear leukocyte infiltration. Arterioscler Thromb Vasc Biol.

[46] Zhang YL, Cao HJ, Han X, Teng F, Chen C, Yang J, Yan X, Li PB, Liu Y, Xia YL (2020). Chemokine receptor CXCR-2 initiates atrial fibrillation by triggering monocyte mobilization in mice. Hypertension.

[47] Zhang YL, Teng F, Han X, Li PB, Yan X, Guo SB, Li HH (2020). Selective blocking of CXCR2 prevents and reverses atrial fibrillation in spontaneously hypertensive rats. J Cell Mol Med.

[48] Ye D, Yang K, Zang S, Lin Z, Chau HT, Wang Y, Zhang J, Shi J, Xu A, Lin S, Wang Y (2016). Lipocalin-2 mediates non-alcoholic steatohepatitis by promoting neutrophil-macrophage crosstalk via the induction of CXCR2. J Hepatol.

[49] Kazankov K, Jorgensen SMD, Thomsen KL, Moller HJ, Vilstrup H, George J, Schuppan D, Gronbaek H (2019). The role of macrophages in nonalcoholic fatty liver disease and nonalcoholic steatohepatitis. Nat Rev Gastroenterol Hepatol.

[50] Wang T, Sun G, Wang Y, Li S, Zhao X, Zhang C, Jin H, Tian D, Liu K, Shi W (2019). The immunoregulatory effects of CD8 T-cell-derived perforin on diet-induced nonalcoholic steatohepatitis. FASEB J.

[51] Mukai K, Miyagi T, Nishio K, Yokoyama Y, Yoshioka T, Saito Y, Tanaka S, Shigekawa M, Nawa T, Hikita H (2016). S100A8 production in CXCR2-expressing CD11b+Gr-1high cells aggravates hepatitis in mice fed a high-fat and high-cholesterol diet. J Immunol.

[52] Hu YF, Chen YJ, Lin YJ, Chen SA (2015). Inflammation and the pathogenesis of atrial fibrillation. Nat Rev Cardiol.

[53] Aschar-Sobbi R, Izaddoustdar F, Korogyi AS, Wang Q, Farman GP, Yang F, Yang W, Dorian D, Simpson JA, Tuomi JM (2015). Increased atrial arrhythmia susceptibility induced by intense endurance exercise in mice requires TNFalpha. Nat Commun.

[54] Liu X, Wang Y, Ming Y, Song Y, Zhang J, Chen X, Zeng M, Mao Y (2015). S100A9: a potential biomarker for the progression of non-alcoholic fatty liver disease and the diagnosis of non-alcoholic steatohepatitis. PLoS ONE.

[55] Krenkel O, Hundertmark J, Abdallah AT, Kohlhepp M, Puengel T, Roth T, Branco DPP, Mossanen JC, Luedde T, Trautwein C (2020). Myeloid cells in liver and bone marrow acquire a functionally distinct inflammatory phenotype during obesity-related steatohepatitis. Gut.

[56] Muise AM, Xu W, Guo CH, Walters TD, Wolters VM, Fattouh R, Lam GY, Hu P, Murchie R, Sherlock M (2012). NADPH oxidase complex and IBD candidate gene studies: identification of a rare variant in NCF2 that results in reduced binding to RAC2. Gut.

[57] Garcia-Jaramillo M, Spooner MH, Lohr CV, Wong CP, Zhang W, Jump DB (2019). Lipidomic and transcriptomic analysis of western diet-induced nonalcoholic steatohepatitis (NASH) in female Ldlr -/- mice. PLoS ONE.

[58] T-cell dysfunction limits immunotherapy efficacy in NASH-induced liver cancer**.** Cancer Discov 2021; 11:OF31. 10.1158/2159-8290.CD-RW2021-047 33811045

[59] Cai J, Xu M, Zhang X, Li H (2019). Innate immune signaling in nonalcoholic fatty liver disease and cardiovascular diseases. Annu Rev Pathol.

[60] Verhelst K, Carpentier I, Beyaert R (2011). Regulation of TNF-induced NF-kappaB activation by different cytoplasmic ubiquitination events. Cytokine Growth Factor Rev.

[61] Qu YC, Du YM, Wu SL, Chen QX, Wu HL, Zhou SF (2009). Activated nuclear factor-kappaB and increased tumor necrosis factor-alpha in atrial tissue of atrial fibrillation. Scand Cardiovasc J.

[62] Ren M, Li X, Hao L, Zhong J (2015). Role of tumor necrosis factor alpha in the pathogenesis of atrial fibrillation: a novel potential therapeutic target?. Ann Med.

[63] Solt LA, May MJ (2008). The IkappaB kinase complex: master regulator of NF-kappaB signaling. Immunol Res.

[64] Liu M, Gu L, Sulkin MS, Liu H, Jeong EM, Greener I, Xie A, Efimov IR, Dudley SC (2013). Mitochondrial dysfunction causing cardiac sodium channel downregulation in cardiomyopathy. J Mol Cell Cardiol.

[65] Zhao CY, Yan L, Wang YD, Wang W, Zhou JY, Zhen Z (2009). Role of resistin in inflammation of hepatocytes in nonalcoholic steatohepatitis. Zhonghua Gan Zang Bing Za Zhi.

