# Supplementary material for: Identification of genes and key pathways underlying the pathophysiological association between nonalcoholic fatty liver disease and atrial fibrillation
Source: BMC Med Genomics. 2022 Jul 5;15:150. doi: 10.1186/s12920-022-01300-1 (PMC9258143; doi:10.1186/s12920-022-01300-1)
Supplement: Supplementary file 7 — Additional file 7: Table S1. Forty-five common genes of NAFLD and AF. [file 12920_2022_1300_MOESM7_ESM.docx]

Supplementary Table 1

Forty-five common genes of NAFLD and AF.

|  | DEGs | Gene name |
| --- | --- | --- |
| AF | Upregulated genes | S100A12, CXCR2, MNDA, S100A8, CLC, GPR160, C16orf54, P4HA1, PKP2, SLC6A6, ARRDC3, EVI2B, IFI44L, HLA-DRA, NCF2, CMTM2, LRRK2, RBM3, CLEC4A, CCR2, LMBR1, MPEG1, FMO2, CHIC2, NEURL1B, FPR3, S100A9, CLEC2B, IFI16, CSF2RB, PTPRC, LRCH1, COL6A3, SYNE2 |
|  | Downregulated genes | SLC2A3, SLC51A, SLC22A4, SLC41A2, BCHE, STC1  IER3, CCL20, CXCL1, SLPI, PRG4 |
| NAFLD | Upregulated genes | CCL20, S100A12, S100A8, MNDA, S100A9, CXCR2  NCF2, CSF2RB, HLA-DRA, CMTM2, IER3, CLC, RBM3, ARRDC3, LRRK2, PTPRC, EVI2B, CLEC2B, BCHE, C16orf54, MPEG1, SLC2A3, IFI16, PRG4, CXCL1, IFI44L, SLC6A6, GPR160, LMBR1, FPR3  CHIC2, PKP2, SLC22A4, SYNE2, LRCH1, SLC41A2  CLEC4A, CCR2, STC1 |
|  | Downregulated genes | COL6A3, SLPI, NEURL1B, FMO2, SLC51A, P4HA1 |

AF, atrial fibrillation; NAFLD, nonalcoholic fatty liver disease; DEGs, differentially expressed genes.
