# Supplementary material for: Identification of genes and key pathways underlying the pathophysiological association between nonalcoholic fatty liver disease and atrial fibrillation
Source: BMC Med Genomics. 2022 Jul 5;15:150. doi: 10.1186/s12920-022-01300-1 (PMC9258143; doi:10.1186/s12920-022-01300-1)
Supplement: Supplementary file 8 — Additional file 8: Table S2. Primer Sequences for qRT-PCR. [file 12920_2022_1300_MOESM8_ESM.docx]

Supplementary Table 2

Primer Sequences for qRT-PCR.

| Gene Names | Forward (5’-3’) | Reverse (3’-5’) |
| --- | --- | --- |
| CCR2 | TCATCTATGCCTTCGTTGGGG | AGGCGTGTTTGTTGAAGTCAC |
| PTPRC | ACCACAAGTTTACTAACGCAAGT | TTTGAGGGGGATTCCAGGTAAT |
| CXCR2 | TCTACCCTGCCCCCTTTTCTAC | GCAAGGTCAGGGCAAAGAGTA |
| MNDA | CCCACTACCCCAGACCTCAT | TGGGTCGTTTTGGGGAACAT |
| S100A9 | CATGGAGGACCTGGACACAAA | CCCTCGTGCATCTTCTCGTG |
| NCF2 | AAGCTGTTTGCCTGTGAGGT | AGACACACTCCATCGCCTTG |
| S100A12 | CCAAGGCCTGGATGCTAATCA | CTTTGTGGGTGTGGTAATGGG |
| S100A8 | GTCTACCACAAGTACTCCCTGAT | TTTGTGGCTTTCTTCATGGCTTT |
